# Ultrafast Dynamics of Charge Transfer and Photochemical Reactions in Solar Energy Conversion

**DOI:** 10.1002/advs.201800221

**Published:** 2018-10-11

**Authors:** Jing‐Yin Xu, Xin Tong, Peng Yu, Gideon Evans Wenya, Thomas McGrath, Matthew James Fong, Jiang Wu, Zhiming M. Wang

**Affiliations:** ^1^ Institute of Fundamental and Frontier Sciences University of Electronic Science and Technology of China Chengdu 610054 P. R. China; ^2^ Department of Physics Lancaster University Lancaster Lancashire LA14YW UK; ^3^ Department of Electronic and Electrical Engineering University College London Torrington Place London WC1E7JE UK

**Keywords:** energy transfer dynamics, photocatalysis, solar energy conversion, time‐resolved spectroscopy

## Abstract

For decades, ultrafast time‐resolved spectroscopy has found its way into an increasing number of applications. It has become a vital technique to investigate energy conversion processes and charge transfer dynamics in optoelectronic systems such as solar cells and solar‐driven photocatalytic applications. The understanding of charge transfer and photochemical reactions can help optimize and improve the performance of relevant devices with solar energy conversion processes. Here, the fundamental principles of photochemical and photophysical processes in photoinduced reactions, in which the fundamental charge carrier dynamic processes include interfacial electron transfer, singlet excitons, triplet excitons, excitons fission, and recombination, are reviewed. Transient absorption (TA) spectroscopy techniques provide a good understanding of the energy/electron transfer processes. These processes, including excited state generation and interfacial energy/electron transfer, are dominate constituents of solar energy conversion applications, for example, dye‐sensitized solar cells and photocatalysis. An outlook for intrinsic electron/energy transfer dynamics via TA spectroscopic characterization is provided, establishing a foundation for the rational design of solar energy conversion devices.

## 1 Introduction

Over the last several decades, the higher capital cost of photovoltaic technologies as compared to fossil fuels has been a major barrier to large‐scale deployment of solar energy,^1^ which has the potential of supplying a non‐negligible fraction of our energy needs.^2^ In this perspective, it is significant to investigate the chemical/physical processes in solar energy conversion so as to enhance its efficiency and lower the overall cost from the design and synthesis of optoelectronic materials to photovoltaic applications.^1^, ^3^ For the typical applications of solar energy conversion, solar cells and solar‐driven photocatalysis‐based systems have attracted a large number of research interests in the last few years.^4^ In these solar energy harvesting processes, photoelectron transfer inevitably occurs in these processes of solar energy conversion.^5^

Due to the fact that the transfer of electron energy is an ultrafast kinetic process in the timescale from femtoseconds (fs) to the millisecond (ms), it is important to develop a powerful characterization technique to observe the transient dynamics in this process. In 1950, the pioneering flash photolysis technique was developed by R. G. W. Norrish and G. Porter,^6^ which was recognized in 1967 by the Nobel Prize in Chemistry. In their experiments, a burst of light with ms duration from discharge lamps was used to initiate chemical reactions.^6^ The time‐resolved spectroscopic methods for the study of charge carrier in dynamics were then developed in the early studies of Zewail, being awarded the Nobel Prize in Chemistry in 1999. The time‐resolved spectroscopy is possible to observe the atomic‐scale dynamic of the chemical bond in a molecule during a chemical reaction with rapid laser excitation.^7^ This technique has been widely applied to investigate the chemical reactions including the broken bond of diatomic molecules and dynamics in large macromolecule and biological molecules.^8^ These time‐resolved spectroscopic methods have made worldwide breakthroughs in the comprehension of fundamental photochemical processes.[[qv: 8b]] Time‐resolved spectroscopic methods can provide the quantitative and qualitative determination of the kinetics of intermediate states in photoinduced chemical reactions by changing the experimental parameters including time resolution, maximum pump‐probe delay, and spectral probe detection bandwidth.^9^

**Figure 1:**
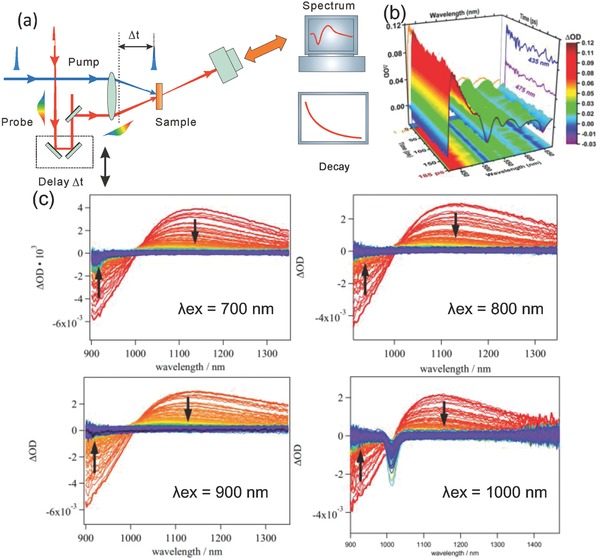
a) Simplified scheme of ultrafast spectroscopy method: pump‐probe technique. b) The 3D surface plot of TA spectra of porphyrins on the absorptions wavelength excitation. Reproduced with permission.^15^ Copyright 2015, American Chemical Society. c) TA signals in the NIR region from 900 to 1350 nm at various time delays from *t* = 0 (red) to *t* = 50 ps (purple) using different excitation wavelengths. Reproduced with permission.^17^ Copyright 1979, American Chemical Society.

Among various time‐resolved spectroscopic methods, ultrafast time‐resolved spectroscopy has become a vital technique as the timescale of the majority of photochemical reactions and photoelectronic transfer processes range from picosecond (ps) to nanosecond (ns).^10^ Ultrafast TA spectroscopy is one of the most commonly used ultrafast time‐resolved spectroscopy for the investigation of the energy transfer and charge carrier dynamics in photochemical systems.^11^, ^12^ **Figure** **1**a presents a schematic diagram of an ultrafast TA spectroscopy measurement. Generally, the absorbance of the sample at a specified wavelength or a range of wavelengths is measured as a function of time after the excitation via a pulsed light. In a typical experiment, both the light for excitation (pump) and the light for measuring the absorbance (probe) are generated by a pulsed laser. The crucial issue for the measurement of diverse timescale is the precise control of the transient delay time between pump and probe pulses. The timescale ranging from ns scope to µs or more can be easily accessible by the electronic control of the temporal pump‐probe delay. However, it is impractical to obtain the pump‐probe delay on timescale below 100 ps via the electronic control due to the physical limitations.[[qv: 8b]] It is realizable to integrate the experimental technique of transient‐absorption spectroscopy with time scale of ns‐µs into an existing instrument for transient‐absorption measurement with timescale of femtosecond (fs) to ps. In such transient‐absorption experiment, the sample is excited by the intense pulse of the pump source and then probed with a broadband white light pulsed at a delay time Δ*t*. The detected light from probe source passes through the sample to a monochromator/spectrograph and is then measured by a detector. The TA properties of the sample before, during, and after the excited pulse are converted by the detector into electronic signals that are displayed in the oscilloscope or charge coupled device (CCD) camera. The timescale ranges from ns to µs or even milliseconds (ms) can be provided by electronic delay triggering.^13^ On the other hand, the temporal Δ*t* values of timescale from fs to ns are provided by a computer‐controlled mechanical delay stage to control the optical path length of the pump or probe pulse, that is, the optical pathway of 3 µm corresponds to a temporal delay of 10 fs.^13^, ^14^

The TA properties are generally defined as the variation of optical density. The units of optical density can be defined according to the following Equation (1)

(1) $$\Delta \mathrm{OD}\left(t,\lambda \right)=\log\frac{I_{100}}{I\left(t,\lambda \right)}$$

where *I*_100_ is the light intensity measured through the sample before generation of excited states and *I*(*t*,λ) is the light intensity measured from the sample after being excited.

Normally, in the case that ignore the singlet–singlet transitions, the detailed changes of optical density can be summarized as follows (2)

(2) $$\Delta \mathrm{OD}\left(t,\lambda \right)=\varepsilon_{T}\;\left(\lambda \right)c_{T}\left(t\right)d-\varepsilon_{G}\left(\lambda \right)\left[c_{T}\left(t\right)+c_{S}\left(t\right)\right]d-\Phi \left(\lambda \right)c_{S}\left(t\right)$$

where ε_G_ is the extinction of the ground state and ε_T_ is the extinction of the transient, *c*_T_ and *c*_S_ are the concentrations of excited triplet and singlet species, respectively, *d* is the effective optical path length of the probe beam, and Φ is the fluorescence spectrum of the sample. As shown in Equation (2), if measurements are made in regions where ground‐state absorption is predominant, the ΔOD is negative, while the decay is often characterized by identical decay kinetics (*c*_S_ = 0). In the case with no sample ground‐state absorption (ε_G_ = 0) and no fluorescence (Φ = 0), the true triplet–triplet features are revealed by the observable ΔOD, and the ΔOD is positive.

For pump‐probe TA technique, the light is generally generated from the pulsed laser. The wavelength of the laser source to excite the photoelectric materials is determined by their optical properties (i.e., absorption spectra). As shown in Figure 1b, the TA spectra of the pump‐probe characterization can show the dynamics of excited states in the samples.^9^, ^15^, ^16^ Benefitted from the development of laser techniques, the ultrafast optical pump‐probe techniques are available with tunable wavelengths. If using the laser source with different wavelengths to irradiate the photoelectric material, the shapes of the resulting TA signals are generally independent of the excitation wavelength. However, the decay lifetime of the excited states may be different as observed via the kinetic plots of the TA spectra. For instance, Gluyas et al. investigated the TA spectra of [Ru(PPh_3_)_2_(µ‐C≡CC≡C)]^+^ in solution with different excitation wavelengths at 700, 800, 900, and 1000 nm (Figure 1c).^17^ As‐measured TA spectra exhibit two types of excited‐state decay: the shorter lifetime which can be recognized as the ground state recovery process and the longer lifetime due to the vibration of the bridge between atoms. This type of vibration based on the high‐frequency ν(C≡CC≡C) mode usually acts as an energy acceptor of the excited states.^18^

In addition, there are other ultrafast optical techniques for measuring time‐resolved spectroscopies such as multidimensional nonlinear spectroscopy and extreme ultraviolet reflection‐absorption (XUV‐RA) spectroscopy. The information of the vibration between the atoms and molecules can be investigated via multidimensional ultrafast spectroscopy, which is a powerful method to obtain the details of the vibrational decays and intramolecular redistributions. The multidimensional ultrafast spectroscopy is also highly sensitive to the information among the molecules such as vibration of bridging ligands and molecule–solvent interactions.^19^ Furthermore, the information of oxidation states, spin states, and ligand coordination geometry for the electronic structure characterization of optomaterials can be provided via XUV‐RA spectroscopy, which may open many exhilarating opportunities with respect to exhaustive studies of electron dynamics in energy conversion materials.^20^

As mentioned above, the TA spectroscopic method provides a direct insight into the fundamental principle of photochemical and photophysical processes. Similarly, there are lots of photochemical reactions during the solar energy conversion processes from the design, synthesis, and applications of optoelectronic materials. Photocatalysis and photovoltaic devices have attracted broad interests since they are promising applications for solving the energy crisis by using solar energy.^21^ The mounting investigations of organic and inorganic semiconductors on the applications of solar energy conversion include transforming or storing photon energy into chemical bonds and electronic energy,^22^ such as photocatalytic hydrogen production, reduction of carbon dioxide, and photochemical synthesis.^23^ Although these approaches have demonstrated excellent potentials in solar energy conversion, the important photophysical and photochemical processes in these systems have yet to be explained in order to realize efficient photochemical conversion, examples include excited states adsorption and reactions on charge/energy transfer processes.[[qv: 8b,24]] Here, we will briefly discuss the fundamental photoexcitation and electron transfer processes occurring on several optoelectronic materials by using TA spectroscopy technique. As these materials and photoinduced processes were involved in solar energy conversion applications such as photocatalysis and photovoltaic devices, we will also demonstrate that the TA spectroscopy technique can provide direct and unambiguous observation of charge and electron transfer dynamics.

## 2 Types of Excited States and Energy Transfer Processes

**Figure 2:**
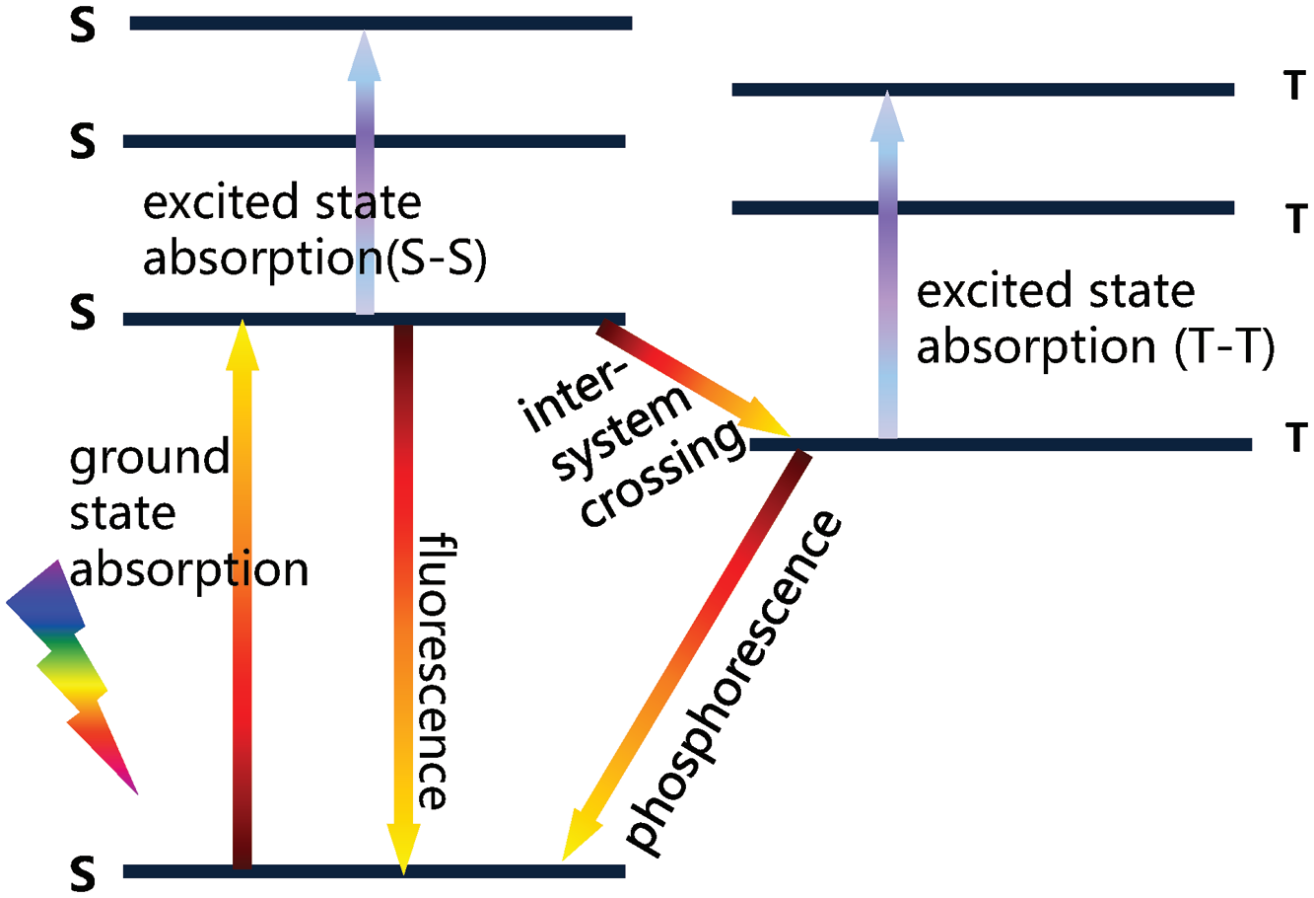
The scheme of energy level showing excited‐state absorption.

Ultrafast TA spectroscopic measurement is able to uncover the fundamental photophysical and photochemical processes in solar energy conversion applications and photochemical reactions.^25^ The photoinduced excited states and electron/energy transfer are constituents of the dynamic processes in photochemical reactions.^26^ Understanding the excited states generation and electron transfer kinetics of photoelectric materials by TA spectroscopic technique is helpful to develop their applications.^27^ As‐measured samples are generally excited by optical stimulation in TA spectroscopic measurement, so that the powerful excitation source such as a laser is usually used in order to generate a sufficiently high concentration of excited states. The excited states can be categorized into the short‐lived excited state (from ps to ns) and long‐lived excited state species (from ns to ms).^28^ The short‐lived excited states are usually associated with singlet–singlet transitions. As shown in **Figure** **2**, the singlet excited is often rapidly quenched via fluorescence and other processes (e.g., intersystem crossing) since there is no more time left for those species to undergo further absorption into higher singlet states. The long‐lived triplet–triplet state is often limited by spin‐forbidding, so transitions from triplet state to singlet state are impossible in this case. The generation of triplet states is attributed to intersystem crossing which is long‐lived and possible to transfer into higher triplet states because of the spin‐allowance feature. Therefore, the excitation of energy‐transfer processes can be described as Figure 2: singlet–singlet energy transfer (SSET),^29^ triplet–triplet energy transfer (TTET),^30^ and triplet–triplet annihilation (TTA).[[qv: 30a,b,d]] Here, TTA is a process in which two triplets are converted into a singlet. All of these energy transfer processes in excited states are classified into Förster resonance energy transfer (FRET)^31^ and/or electron transfer.^32^

Theoretical calculation of these excited states and energy transfer processes in photoelectric materials have been conducted by an increasing number of works.^33^ It is still inevitable to consider the basic principle of photochemical and photophysical processes in terms of experimental measurement.[[qv: 32b,34]] The generation of several excited states and the types of spin‐allowed electron/energy transfer processes have been discussed in detail. All of the generation, annihilation, and transmission of these excited states can be directly observed via TA spectroscopy technique. These initial photoinduced processes based on photoelectric materials and photochemical reactions are significant for clearing the challenges of the solar energy conversion and photovoltaic applications.

## 3 Interfacial Charge Transfer Dynamics in Photoelectric Materials

The basic photochemical and photophysical processes occur during the photoexcitation of photoelectric materials and many promising materials have been developed for solar energy conversion applications.^35^ However, the major challenges still remain to link the fundamental photoinduced processes with the overall application and device performance.^36^ Further efforts should be made to develop experimental techniques to investigate the excited‐state properties of the photoelectric materials. The properties of photoelectric materials such as optoelectronic organic molecules and nanostructured materials can be observed in situ and directly via TA spectroscopy technique.^37^

Interfacial charge transfer often occurs on nanostructured materials due to their high intensity of interfaces.^38^ This process usually consists of exciton generation and transfer and has drawn numerous attention due to its great potential to break Schockley–Queisser (SQ) limit in fundamental procedures of energy conversion.^39^ All of these optoelectronic materials including semiconducting quantum dots (QDs),^40^ 2D and 3D nanostructures^41^ are extremely promising to be used as photofunctional materials due to their size‐tunable electronic and optical properties.[[qv: 36b,42]] The excited excitons in semiconductor materials could be transferred among the bulk solution and/or between each other through their interfaces, which has tremendous potential in a range of chemical transformations applications such as optoelectronics, photobiology, and solar energy conversion.^43^ For these application systems, the photoexcited excitons can act as both electron donor and acceptor.^44^

**Figure 3:**
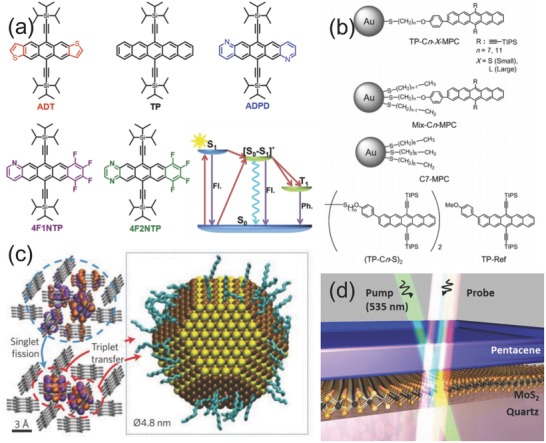
a) Molecular structures of pentacene derivative and illustration of the SF mechanism in solutions: a nearby ground‐state molecule forms an excimer [S_0_‐S_1_]*, then the excimer intermediate decays to two triplets. Reproduced with permission.[[qv: 37c]] Copyright 2016, American Chemical Society. b) The chemical structures of pentacene self‐assembled monolayers on gold nanoparticles. Reproduced with permission.^51^ Copyright 2009, John Wiley and Sons. c) Schematic of triplet exciton transfer from tetracene to a PbS nanocrystal with decanoic acid ligands. Singlet excitons in tetracene first undergo singlet fission then triplet transfer to the nanocrystal. Reproduced with permission.[[qv: 50a]] Copyright 2014, Nature Publishing Group. d) Schematic of monolayer MoS_2_‐pentacene van der Waals heterojunction probed by TA spectroscopy. Reproduced with permission.^41^ Copyright 2017, American Chemical Society.

Organic semiconductor materials such as small organic semiconductor molecules have exhibited singlet (S_1_) and triplet states (T_1_)^45^ that are promising to be applied in energy/electron transfer devices.^46^ The singlet state process generally has a short‐lived timescale, while the triplet state possesses a long‐lived excited timescale. The short‐lived excited states do not have enough transport time for energy conversion, as short‐lived states result in a very rapid decay. These excited states of optoelectronic materials, pentacene and its derivatives, are famous in high‐efficiency harvesting of excited states among optoelectronic organic molecules because of their unique property: singlet exciton fission (SF).[[qv: 8a,47]] As shown in **Figure** **3**a, the process of SF can be briefly described as below: a molecule absorbs one photon at a corresponding energy level and then transforms from the ground state to a singlet excited state. The singlet state then interacts with the surrounding molecule and generates a steady intermediate excimer which can decay to form two triplet excitons. These long‐lived triplet states can be efficiently transferred to targets which are used for energy conversion applications.^48^ Many efforts have been devoted to acquiring a high yield of triplet excited states, such as design of structures or suitable chromophores based on pentacene,[[qv: 37c,49]] tunable heterojunction,^41^ and constructing a bridge between donors and acceptors.^50^ Figure 3a depicted a series of pentacene and its derivatives with the triplet yield of more than 100% from SF, which have been reported by Zhang et al.[[qv: 37c]] The singlet excitons rapidly decay to long‐lived triplet excitons are directly evidenced by TA spectroscopic measurement.[[qv: 37c]] To achieve efficient generation of excited triplet states via SF, Kato et al. have recently designed a pentacene‐based heterostructure (Figure 3b), in which the gold nanoparticle and alkane chain possess different sizes and lengths, respectively. The generation of triplet states at a high yield approaching 172% was observed by time‐resolved TA measurement.^51^ As shown in Figure 3c, a breakthrough in the near‐infrared‐harvesting triplet states has been realized by Thompson et al. The triplet states were generated from the organic semiconductor tetracene via SF, and then the triplet excitonic energy transfer from tetracene to PbS nanocrystals process was directly demonstrated by TA spectra studies.[[qv: 50a]] Furthermore, the high efficiency of triplet excitons applied in photovoltaic devices has been reported. The group of Weiss and co‐workers demonstrated van der Waals heterojunctions between p‐type pentacene (0D) and n‐type monolayer MoS_2_ (2D) for photovoltaic applications (Figure 3d). The kinetics of the excited carriers in the system were studied via TA spectroscopy.^41^ These transfer dynamics of excited states in nanostructured materials and organic molecules on a transient timescale offer a direct understanding of optoelectronic properties. The advanced TA spectroscopic characterization plays a central role in revealing the properties, limitations, and ultrafast electron dynamics in the optoelectronic material.

**Figure 4:**
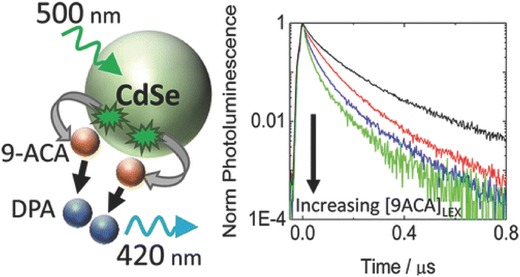
Dynamics of energy transfer from CdSe nanocrystals to triplet states of anthracene ligand molecules in a hybrid system. Reproduced with permission.^56^ Copyright 2016, American Chemical Society.

Unlike organic molecules with distinct singlet and triplet states, the photoexcitation of typical semiconductor QDs has complex, multiexcited states.[[qv: 10b]] This means that when the QDs are excited, diverse energy levels of excited states can be produced. The multiple excited states are favorable to be applied in various donor–acceptor systems for electron or energy transfer. Several investigations have demonstrated the transformation of multiple excited states from QDs, in order to realize the diversified level of energy/electron transfer by single or multiple electronic absorptions.[[qv: 25a,28a,40a,52]] Energy transfer from excited states of semiconductor nanocrystals to molecular singlet states has been widely investigated.^53^ In these studies, semiconductor QD‐organic semiconductor molecules hybrid systems were constructed, which have unique optical and electronic properties.[[qv: 40c,54]] These include nanocrystals absorbing the low energy level photons and then the transferring the energy to the acceptors, usually the molecular triplet states. In order to achieve photon upconversion across the visible and near‐infrared region, Huang et al. constructed a hybrid system which combined CdSe and PbSe QDs with molecular emitters.^55^ This work presented the photon upconversion at the wavelength of 532 and 420 nm. Moreover, understanding the kinetics of the energy transfer from semiconductor nanocrystals to acceptors can guide further developments toward the tunable excitation and emission wavelengths. As shown in **Figure** **4**, energy transfer in a hybrid system with CdSe nanocrystals combined with the ligand binding of 9‐anthracene carboxylic acid (9‐ACA) was demonstrated via time‐resolved PL measurements.^56^ The time‐resolved PL shows the long‐lived PL properties in the case that increasing the 9‐ACA which acts as the excited energy acceptor.

**Figure 5:**
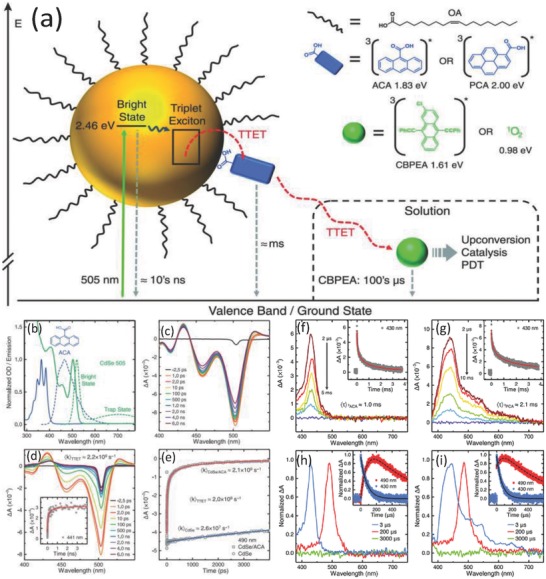
Direct investigation of nanocrystal‐to‐solution triplet energy transfer and decay pathways. a) Kinetic profiles and b–i) quenching studies of ACA and PCA triplet state populated from excited CdSe nanocrystals. Reproduced with permission.[[qv: 27b]] Copyright 2016, the American Association for the Advancement of Science.

The dynamic of triplet state transfer from semiconductor nanocrystals was also reported by Mongin et al.[[qv: 27b]] The process of triplet energy transfer and decay pathways from nanocrystal to solution are schematically illustrated in **Figure** **5**a. The CdSe semiconductor nanoparticles were excited by green light at the wavelength of 505 nm to generate exciton states. On the surface of the nanocrystal, triplet states were transferred from semiconductor nanocrystals to the targeted polyaromatic carboxylic acid acceptors. This process can be described as interfacial Dexter‐like triplet–triplet energy transfer.^57^

The direct observation of kinetic transfer process of triplet energy to acceptor was investigated by TA spectroscopy. As shown in Figure 5c,d, TA spectroscopy measurement was performed on CdSe‐oleic acid (OA) in toluene and CdSe‐OA/anthracene carboxylic acid (ACA) hybrid system with triplet acceptor. It was demonstrated that the triplet–triplet energy transfer occurs when the surface‐anchored ACA (acceptor) exists, since the CdSe excited state at 433 nm decayed in 2 ns and new absorption observed at 441 nm was attributed to triplet ACA (3ACA*).[[qv: 57a]] The transient kinetic parameters of TTET from semiconductor nanocrystals to ACA can then be analyzed by TA spectroscopy measurement (Figure 5e). Identically, the kinetics of CdSe/ACA and CdSe/1‐pyrone carboxylic acid (PCA) with a secondary acceptor originated from TTET were extracted by TA spectroscopy measurements (Figure 5f,g).^58^ The electron transfer rates between the semiconductor and different excited states species can be carried out according to their TA decay kinetics (Figure 5h,i) at corresponding excitation wavelength.

Colloidal semiconductor QDs exhibit the wide absorption and PL spectra ranging from ultraviolet–visible to near‐infrared region due to their size/chemical composition‐dependent optoelectronic properties, which make them become the promising building blocks for solar technologies.[[qv: 4c,59]] Over the past decades, various types of QDs with excellent optical and electrical properties have attracted lots of research interests and leads to a rapid development in this field.^60^ The excited charge transfer dynamics of bare QDs (e.g., perovskite, CdS, and CdSe QDs), which are typically overlaid with surface ligands and exhibit efficient photoinduced charge carriers, have been discussed in the above section. The photoinduced excitation transfer from the QDs to the surficial acceptor or surroundings is a type of charge transfer dynamics in QDs system, which reveal their fundamental optoelectronic properties to facilitate the improvements of QDs‐based applications.^61^ However, these bare QDs still have major limitations such as the high surface sensitivity that leads to the formation of surface‐related trap states.[[qv: 60a,62]]

**Figure 6:**
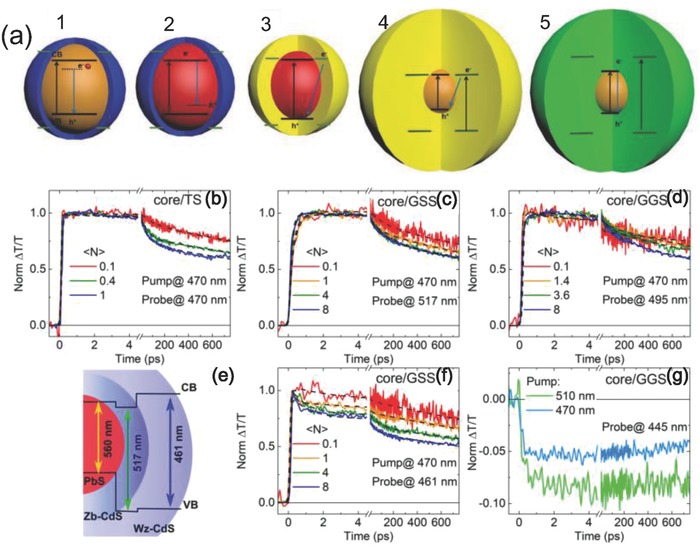
a) Different types of core/shell structure QDs. Reproduced with permission.^64^ Copyright 2018, The Royal Society of Chemistry. Normalized TA spectrum of core/shell QDs with different structures: b) core/thin shell (core/TS), c) core/giant sharp shell (core/GSS), d) core/giant graded shell (core/GGS), f) core/GSS, and g) core/GGS. e) The scheme of band alignment of core/GSS nanocrystals. Reproduced with permission.^65^ Copyright 2017, American Physical Society.

The formation of core/shell structured QDs is an effective method to suppress the surface trap states due to that the inorganic shell on core QDs is able to prevent the interaction between core QDs with the ambient environment, thus improving the surface passivation.^63^ Furthermore, because of the tunable thickness and components of shell materials, the core/shell QDs are able to exhibit excellent optical properties including tunable absorption/emission spectra, high PL quantum yield. These optical properties are derived from the photoinduced charge carrier dynamics in the core/shell QDs systems that excited electron/hole separation and recombination. Zhou et al. reported five types of core/shell QDs systems (**Figure** **6**a) synthesized via different approaches. In the first QDs system, the photoexcited state could be generated from doped cation (such as Mn^2+^) and the rate of energy transfer is much faster than the rate of exciton recombination. The second QDs system shows the native intragap emission from the hole‐like defect, leading to a large Stokes shift. The third QDs system is a core/shell structure in which the core is coated by a wide bandgap semiconductor with suitable energy band, thus resulting in the electron delocalization from the core to the shell region. The fourth QDs system is the giant core/shell QDs with the thick shell that increased delocalization of electrons in the whole shell region. The last one is another type of giant core/shell QDs in which the absorption comes from the shell and the emission is originated from the core.

The TA spectroscopy has been demonstrated as an effective technique to show the dynamics of coupling and decoupling of core and shell excitons in different types of core/shell QDs (Figure 6b–g).^66^ For example, in PbS/CdS QDs, the signal of time‐resolved spectra in ns timescale at a probe wavelength of 470 nm is attributed to the photobleaching (PB) of the CdS shell. When changing the probe wavelength to 517 and 461 nm in core/GSS system, the PB peaks of the QDs present the different decay time in the first 1 ps due to the redistribution between the Wz‐CdS and Zb‐CdS (Figure 6c,f). Time‐resolved spectroscopy measurements with different wavelengths of pump‐probe light were carried out to study the core/GGS QDs system. As shown in Figure 6d, the PB of the core/GGS QDs shows a long lifetime up to 3 ns at 496 nm, which is derived from the recombination of excitons. Similar results of long‐lived exciton were observed with a pump wavelength of 470 nm and a probe wavelength of 495 nm (Figure 6g) which is assigned to the trap states of the QDs.

**Figure 7:**
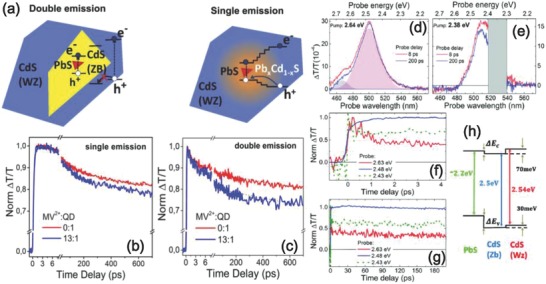
a) A schematic diagram and electronic band structure of double‐emitting and single‐emitting “giant” core/shell QDs. b) TA spectroscopy dynamics for single‐emitting and c) double‐emitting. Reproduced with permission.^68^ Copyright 2016, Elsevier. d–g) TA spectra of PbS/CdS/CdS core/shell/shell QDs with different pump‐probe delays. e) Schematic diagram of energy level in the PbS/CdS core/shell QDs with ZB and WZ phase of CdS shell. Reproduced with permission.^66^ Copyright 2016, The Royal Society of Chemistry.

Furthermore, investigating the ultrafast dynamics of excitons in core/shell QDs can provide insight to understand their basic physics and provide the guideline for the development of QDs‐based optoelectronic devices.[[qv: 25a,44a]] For the last several years, extensive efforts have been made to design and synthesis of core/shell QDs with excellent optical properties based on the understanding of their charge transfer dynamics.[[qv: 61b]] For instance, Rosei's group reported a new type of near‐infrared PbS/CdS core/shell “giant” QDs.[[qv: 62a,66,67]] The interfacial structure of these QDs was designed via tuning the molar ratio of Cd: S in CdS shell grown on the PbS core. In this core/shell QDs, regulation of electron/hole transfer can be controlled by changing the gradient interfacial layer. The tunable interfacial charge transfer can lead to the conversion of excited emission, for example, from double‐color emission to single‐color emission.^65^, ^66^ The absorption spectra of these QDs cover ultraviolet/visible to near‐infrared region, which is promising for solar energy conversion.[[qv: 44a]] The schematic diagram and electronic band structure of single emission and double emission QDs are depicted in **Figure** **7**a.

Ultrafast TA spectroscopy provides a direct insight into the mechanism of single/double‐color emitting QDs (Figure 7b,c). As shown in the schematic diagram of the two types of QDs, the shell with zincblende (ZB) phase acts as “hole‐blocker” due to the large difference in band offsets between ZB and wurtzite (WZ) CdS in the double emission QDs. The further investigation of charge transfer dynamics by the TA spectroscopy is exhibited in Figure 7b,c. With the presence of the electron acceptor methyl viologen (MV^2+^), the lifetime of both single and double emission QDs have a faster decay. The single emission QDs show a longer lifetime than the double emission counterparts. The ultrafast time‐resolved spectroscopy with different excitation photon energies was carried out with the double emission QDs (Figure 7d–g). The appeared photobleaching signal around 490 nm is originated from the excitations from CdS. Due to the different bandgap between core (PbS) and shell (CdS), the PbS core and CdS shell can be excited by the incident photons with energies at 2.64 and 2.38 eV respectively. These TA spectra results reveal the excited charge transfer dynamics in this type of core/shell QDs system, indicating that the excited electrons are delocalized throughout the entire core and shell region.

In conclusion, we have discussed the dynamic processes of interfacial energy/electron transfer based on several photoelectric materials such as small organic molecules and semiconductor nanocrystals. Semiconductors nanostructured materials have tunable broadband light absorption, ranging from visible to the near‐infrared region, and hold promise to be utilized for numerous triplet excited‐state reactions. All of these properties can be applied to photoredox catalysis, photochemical upconversion, singlet oxygen generation, photochemical synthesis, and excited‐state electron transfer.[[qv: 55a,69]] Direct observation of energy/electron transfer from donor to acceptor by TA spectroscopy technique can provide basic guidelines for designing high‐efficiency optoelectronic applications.

## 4 Dynamics of Electron/Energy Transfer in Solar Energy Conversion

### 4.1 Dynamics of Photocatalysis

We have presented the charge transfer dynamics of several photoelectric materials via the TA spectroscopy technique in the above sections. The unique optical properties of these materials have proven excellent potential in photocatalytic applications with solar energy conversion processes.^21^ Meanwhile, the important photophysical and photochemical processes in these applications in order to realize efficient photochemical transformation have yet to be explained. Clarifying the dynamics of photoinduced electron/energy transfer can help recognize the origin of limitations of conversion efficiency.^70^ Ultrafast time‐resolved spectroscopic techniques, which provide a visualized approach to directly measuring the electron/energy transformation in systems of photochemical conversion from solar energy, have been applied extensively over the past decades.[[qv: 5a,71]]

Efforts on photocatalytic hydrogen production trace back to 1972 and the pioneering experiments were conducted by Fujishima and Honda using titanium dioxide (TiO_2_).^72^ Since then, a large number of ensuing works have focused on either reduction or oxidation of solar water‐splitting redox conversion.^73^ The photocatalytic systems for hydrogen production typically consist of a heterogeneous or homogeneous catalyst,^74^ a photosensitizer, an immolated electron donor, and an electron relay. Lehn and Sauvage designed the reduction of hydrion to hydrogen with a system in 1979, which included the following components:^75^ Ru(bpy)_3_^2+^ as the photosensitizer to absorb the visible light; triethanolamine (TEOA) as electron donor, which provided the electrons during the reduction reaction process; a relay species of the Rh(bipy)_3_^3+^ complex as an intermediate storage of electrons by a reduced state to mediate water reduction; the colloidal platinum which promoted the hydrogen generation, as a catalyst. In recent years, increasing improvements have been made for these systems.[[qv: 74a,76]] In particular, excellent light harvesters were investigated to improve the efficiency of the photocatalytic systems. The photoinduced electron transfer processes constitute the building blocks of photochemical reactions in solar energy conversion.^77^

**Figure 8:**
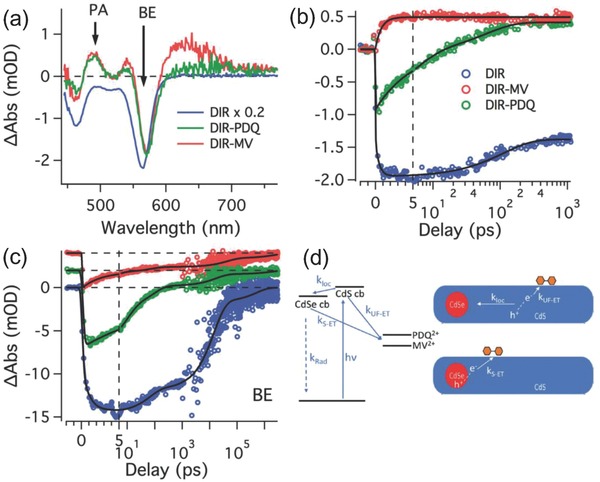
Ultrafast TA of mediator–DIR complexes. a) TA spectra for DIR, DIR–PDQ^2+^, and DIR–MV^2+^ complexes averaged from 800 ps to 1 ns. b) TA of the PA (photoinduced absorption) feature for DIR, DIR–PDQ^2+^ and DIR–MV^2+^ complexes probed at 490 nm. c) TA of the band edge (BE) feature probed at 565 nm. d) Qualitative energy level diagram and schematic representation of the electron transfer processes that occur between photoexcited DIR and mediator. Reproduced with permission.[[qv: 4a]] Copyright 2017, The Royal Society of Chemistry.

Harris and Kamat recently reported the interfacial electron transfer from semiconducting nanostructures (CdSe/CdS dot‐in‐rod (DIR)) to the acceptor in a photocatalytic system. In their work, they provided an insight into the processes of photoconversion and mapped out the electron transfer kinetics, as illustrated in **Figure** **8**d.^78^ DIR structures were constructed to generate the long‐lived charge‐separated states, in which the exciton from photoexcited DIR was extracted rapidly to its surface for redox‐mediated photocatalysis. The photosensitizer can be linked with the photocatalyst via modifications of the redox mediator so that the photocatalytic activity of the system can be prominently improved. TA spectroscopy investigation reveals that the electron transfer from DIR to photosensitizer occurred in both DIR‐MV^2+^ and DIR‐propyl‐bridged 2‐20‐bipyridinium (PDQ^2+^) systems (Figure 8a). There are long‐lived excited states existed at the CdSe/CdS interface and CdSe core. The photoinduced absorption at 600 nm is due to the characteristic absorption of MV^2+^. The corresponding kinetic plots of the TA spectra of DIR‐MV^2+^ and DIR‐ PDQ^2+^ show that electron transfer from DIR to MV^2+^ is faster than the case of PDQ^2+^. Both electron transfer processes are completed within 1 ns (Figure 8b), which are more efficient than the radiative decay process. The dynamics of the TA spectra figuring the band edge (BE) bleaching are shown in Figure 8c. The decay time of DIR‐MV^2+^ is the shortest which is attributed to the direct excited‐state generation in the CdSe, an electron transfer process in the DIR system. Both of the TA dynamics indicate that the charge separated state from photoexcited can exist in 100 ps.

**Figure 9:**
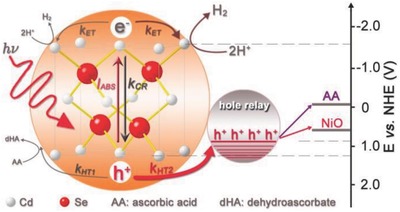
Schematic of representative photoexcited states in the photocatalytic system: The optical excitation produced electrons and holes separation, and the excited states transfer to the acceptor and utilized in photocatalysis. Reproduced with permission.[[qv: 22a]] Copyright 2016, John Wiley and Sons.

Since the early 1990s, it has been recognized that excited states are important to the understanding of charge and energy transfer dynamics.^79^ Wu's group has focused on the important photophysical and photochemical processes: steady‐state and time‐resolved techniques were used to provide the insight that is helpful to achieve efficient photochemical transformation.[[qv: 22b,69a,80]] It is important to understand the exciton energy transfer and charge carrier separation or recombination in the photoinduced reaction. **Figure** **9** shows the schematic diagram of the interfacial charge transfer processes during the photocatalytic hydrogen evolution driven by solar energy.[[qv: 22a]] In this system, the photoinduced electrons and holes can be generated upon the absorption of the light source (*I*_ABS_) by CdSe. The holes transfer from QDs to the surface ligands acting as the hole acceptor (K_HT2_). At the same time, the hole can be accepted by the ascorbic acid in the solution (K_HT1_). Therefore, the photoinduced charge recombination process (K_CR_) can be prevented by these two pathways of photogenerated holes consumption. The long‐lived charge separation and high‐efficiency photocatalytic hydrogen evolution are thus realized.

It is valuable to consider the origin of the excited‐state generation and transformation. The photoinduced electron transfer processes happened both in molecular photocatalysis and heterogeneous photocatalysis systems. The interfacial electron transfer processes are key to driving energy conversion in heterogeneous photocatalysis,^81^ Gao et al. used TA measurements to offer the information of the electron and energy transfer steps of photocatalytic hydrogen evolution, which were based on iron‐doped carbon nitride‐type polymers (2D).^82^

**Figure 10:**
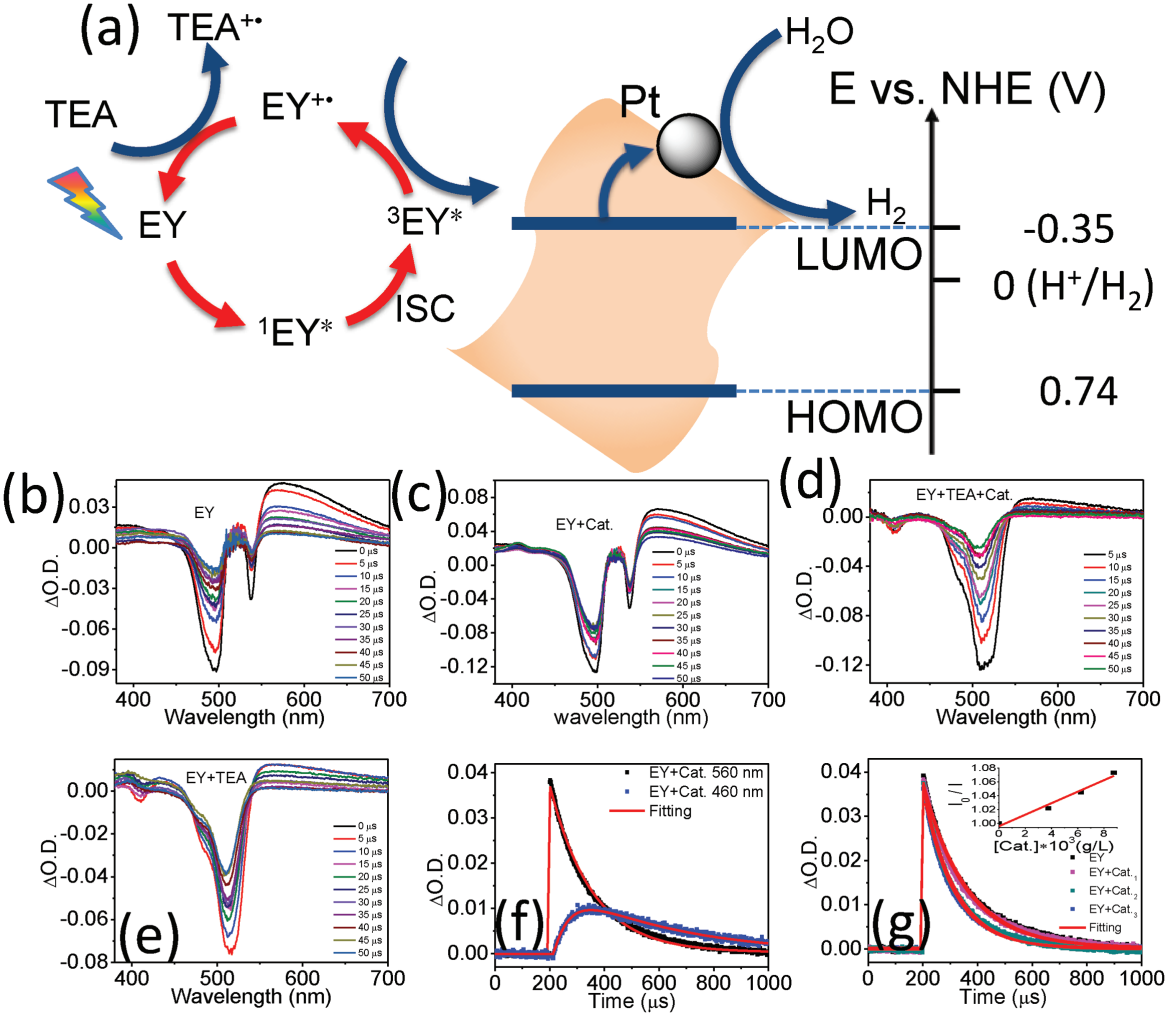
a) Energy and electrons transformation in the photocatalytic system. b–g) The dynamic picture of complex multistep electron transfer during photocatalysis and the influence of charge separated state lifetime on photocatalytic efficiency by time‐resolved technique. Reproduced with permission.^82^ Copyright 2016, American Chemical Society.

As a typical molecular photocatalysis system, the sensitizer and functional catalyst play the role as the main building blocks in the molecular donor–acceptor system. Organic dyes and transition metal complexes are the most widely investigated sensitizers and catalyst in molecular photocatalysis system.^83^ Despite the representative transition metal complexes such as Ru(bpy)_3_^2+^, the photosensitizer of eosin Y has also been extensively studied.^84^ The highly efficient and selective photocatalytic nitrobenzene reduction strategy employing eosin Y as the photocatalyst and triethanolamine (TEOA) as the donor has been reported by Yang et al.[[qv: 80b]] The reaction mechanism was revealed with the understanding of the photoinduced electron transfer processes. The photosensitizer (Eosin Y) exhibited a bleaching of ground state at 520 nm and a TA peak at 560 nm which was recognized to the absorption of excited triplet state of Eosin Y (^3^EY*), with laser excitation at 532 nm.[[qv: 80b]] In addition to nitrobenzene, the absorption at 560 nm synchronously decays when a new absorption region at 460 nm arises. The new absorption at 460 nm is assigned to the radical cation of EY^+^. As shown in **Figure** **10**, the interfacial electron transfer occurs between molecular and nanostructured materials in such photocatalytic systems. In order to study the photocatalytic mechanism of Fe‐g‐CN, the author carried out laser photolysis measurements to understand the dynamic processes during the hydrogen generation. The TA measurements have been taken with EY, a mixture of triethylamine (TEA) and EY and the whole photocatalytic system, respectively. The lifetime components of the samples can be extracted by the transient decay measurements at a single wavelength detected by photomultiplier tube detectors (Figure 10 b–f and **Table** **1**). Least‐squares‐fitting program can be used to fit the probed wavelengths up to four exponentials. The exponential growth or decay process can be expressed by the following mathematical Equation (3)

(3) $$I\left(t\right)=c_{0}+c_{1}e\frac{t}{\tau_{1}}+c_{2}e\frac{t}{\tau_{2}}+c_{3}e\frac{t}{\tau_{3}}+c_{4}e\frac{t}{\tau_{4}}$$

where *I*(*t*) is the decay model referring to the response of the sample to a very short excitation, and *c*_0_ is the background, *c*_1_, *c*_2_, *c*_3_, and *c*_4_ are the pre‐exponential factors determining the weight of characteristic lifetime components of τ_1_, τ_2_, τ_3_, and τ_4_, respectively. The decay and growth processes are indicated by positive pre‐exponential and negative values.

**Table 1:** Tabulated fitting results of lifetime components for the above samples

Sample	403 nm τ (EY^−•^)	460 nm τ (EY^+•^)	560 nm τ (^3^EY*)
EY			159 µs (100%)
EY+Cat.		60 µs (rise, 49%)	154 µs (100%)
		413 µs (51%)	
EY+TEA	56 µs (rise, 55%)		127 µs (100%)
	Long, >ms (45%)		
EY+TEA+Cat	53 µs (rise, 56%)	Long, (>ms)	53 µs (100%)
	Long, >ms (44%)		

Fe‐g‐CN, being a homogeneous catalyst, can be dissolved into this system and the electron transfer rates from EY to Fe‐g‐CN were estimated by the Stern–Volmer plots using the following equation[[qv: 32b,80b]]

(4) $$\frac{I_{0}}{I}=1+k\tau_{0}\left[Q\right]$$

where *I*_0_ is the initial TA intensity of ^3^EY* at 560 nm, *I* is the quenched intensity with increased Fe‐g‐CN concentration of [*Q*], τ_0_ is the lifetime of single ^3^EY*, and *k* is the rate of electron transfer from EY to Fe‐g‐CN. These results proposed the factors that may influence energy and electron transfer in photocatalysis and pave the way for the rational design of highly efficient catalysts. In summary, while the photosensitizers serve to absorb photons and transfer electrons, understanding the detailed processes of the charge carrier dynamics is significant to optimize the photocatalytic and photovoltaic applications.[[qv: 32b,85]] This work has pioneered a description of the electron/energy transfer mechanism of the photocatalytic system by demonstrating the kinetic and photoinduced excitation states and by invoking new intermediates.

### 4.2 Device Constructions of Solar Cells

The performance of photocatalysis has been evaluated based on the investigations of charge transfer kinetics via TA spectroscopy technique. Owing to their potentials of high efficiency in photovoltaic electricity generation, dye‐sensitized solar cells (DSSCs) have emerged to be attractive solar energy conversion devices as well.^86^ The essential properties that control the performance of DSSC are the generation of photocurrent, which is a process involving photoexcitation of the electron and its transfer from the electron donor to electron acceptor.^67^, ^87^ DSSCs are excitonic devices in which the electron transfer across the interface is a primary process that governs the performance of devices including operating lifetimes and efficiencies. The process of electron and energy conversion in DSSC is the dynamic of photoinduced charge transfer (separation and recombination) between the semiconductor, electrolyte, and dye. The TA spectroscopic technique has been employed to detect the transient signals from charged semiconductor nanoparticles and the electronic TA of ionized molecules.^88^

**Figure 11:**
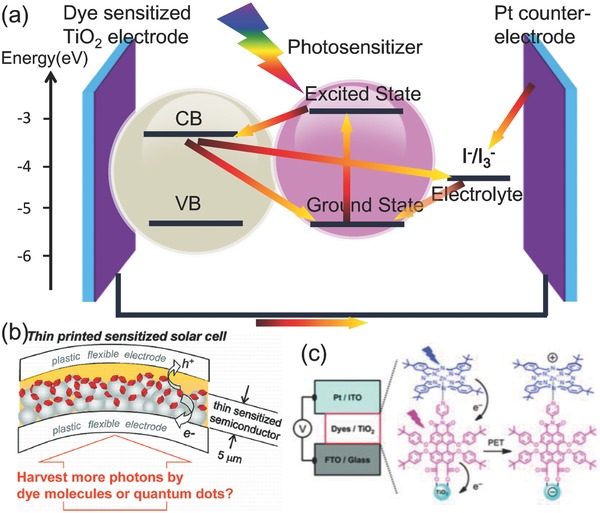
a) Scheme of electron transfer and charge transport processes of DSSC. b) Schematic diagram of the structure of the DSSC. Reproduced with permission.[[qv: 89b]] Copyright 2011, American Chemical Society. c) The electrons from photoexcitation of the sensitizer dye flow into the mesoporous oxide layer and electron transport through the oxide semiconductor to the working electrode. Reproduced with permission.[[qv: 89a]] Copyright 2014, The Royal Society of Chemistry.

**Figure** **11**b,c shows a typical schematic diagram of DSSC.^89^ As the crucial factor of the system, electronic conduction takes place at the mesoporous oxide layer, which is composed of nanometer‐sized particles. The mesoporous oxide layer, from the relatively narrow bandgap materials (e.g., TiO_2_) to wide‐gap oxides (e.g., SnO_2_, Nb_2_O_5_, and ZnO), have all been widely investigated.^90^ The sensitizer of dye which is attached to the surface of the mesoporous oxide layer constitutes the thin sensitized semiconductor film. The extensively studied sensitized dyes include Ru‐bipyridyl dyes, porphyrin compounds, and cyanine dyes.[[qv: 5a,91]] In these systems, electrons from the photoexcited sensitizer are injected into the semiconductor oxide layer, which is defined as the electron donor and acceptor, respectively.^92^ The photoexcitation of the sensitizer dye is returned to ground state by electron contribution from the electrolyte.^93^ All of the electron generation and conduction occurs on the surface of dye sensitizer and oxide semiconductor. Considering the impact of device structures and photonic materials, based on the dynamics of electron transfer, we can learn from such kinetic investigations to further improve device performance.

### 4.3 Charge Carrier Dynamics of Dye‐Sensitized Solar Cells

Early investigations regarding charge transfer were based on the studies of components in DSSC system, such as analyzing the redox potential of different compositions (the dye‐sensitized metal oxide in the nonexistence of redox electrolyte or solar irradiation) by photoelectrochemical measurements.^94^ Although the charge transfer kinetics has made significant progress in exploring a detailed description of the overall device performance, there is still a lack of time‐resolved analysis of charge transfer processes.^95^ Considering that the charge transfer kinetics involved in photovoltaic devices may influence their performance, transient kinetic studies have recently attracted intensive interests.^78^, ^96^

Figure 11a elucidates a photochemical view of the working principle of a DSSC with the sequences of electron transport processes. It also illustrates some competing loss pathways which are indicated by straight arrows of the electron and charge transport processes. These loss pathways include the charge recombination to dye, the excited state of dye decayed to ground state, and recombination to the electrolyte. Similar to natural photosynthesis, kinetic rivalries between the varieties of forward and loss pathways are important to governing the quantum efficiencies of charge separation and recombination, and are therefore critical factors determining energy conversion efficiency. However, the dimension of this injection kinetics based on excited‐state decay to the ground is more essential than the absolute kinetics of electron injection that depended on the efficiency of electron injection in DSSCs. As a typical example, the decay time of the excited‐state varies from photosensitizers to other components, and correspondingly there are different requirements on the kinetics of electron injection that are vital for the high‐performance device.

### 4.4 Investigation of Charge Transfer Dynamics in DSSCs Based on TA Spectroscopy Measurements

The sensitizer of dye is the critical ingredient that governs the performance of the DSSC. The photosensitizer absorbs light and generates excited states, which is then injected into mesoporous oxide semiconductor. The redox electrolytes such as iron complex, triiodide, or cobalt restore the photosensitizer to the ground state by electron donation and transfer electrons to the auxiliary electrode to accomplish the photovoltaic circuit.^97^ However, charge transfer between the injected holes and the photosensitizer occurs on a short timescale of less than milliseconds.[[qv: 94d,98]] Therefore, the dynamic processes of electron transfer are the critical factors to improve the device performance. TA spectroscopy is a time‐resolved technique which can detect transient changes in the absorption of a photoexcited sample to exhibit the excited states generated at a short timescale. In multicomponent systems, different excited states of the absorption signals are broad and overlap. TA spectroscopy is suitable for detecting donor/acceptor systems, in which every element can be preferentially excited.^99^

**Figure 12:**
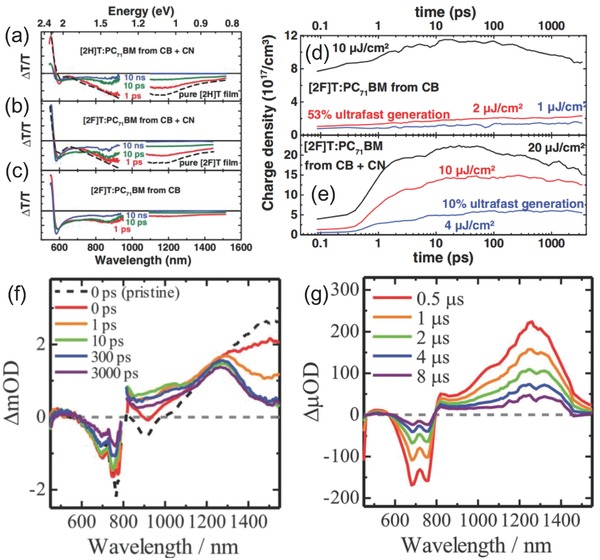
a–c) TA spectra of different polymer donors prepared with and without CN additives; samples excited at 532 nm with 10–11 µJ cm^−2^ pulse^−1^. e,f) The charge carrier density dynamics of [2F]T:PC_71_BM. Reproduced with permission.[[qv: 4b]] Copyright 2014, John Wiley and Sons. f) TA spectra of a PSBTBT/PCBM blend film measured at 0 (red), 1 (orange), 10 (green), 300 (blue), and 3000 ps (purple) after the laser excitation at 800 nm. g) TA spectra of a PSBTBT/PCBM blend film measured at 0.5, 1, 2, 4, and 8 ms after the laser excitation from top to bottom at 400 nm. Reproduced with permission.[[qv: 88c]] Copyright 2014, The Royal Society of Chemistry.

In the work by Julien et al., the TA spectra were used to provide the information of photoexcited states in DSSC.[[qv: 4b]] **Figure** **12**a–c depicts typical TA spectra with time delays of 1 ps, 10 ps, and 10 ns, corresponding to the principal photoinduced states (excitons), the charge generation process, and the residual products after exciton decay and electron transfer. These results recognize that there are no further spectral shift of the photoinduced absorption at longer times, indicating the observed triplet exciton is originated from [2X]T:PC_71_BM (X = H, F) blends. The charge carrier density dynamics of [2F]T:PC_71_BM is shown in Figure 7d,e. The results show that there are two charge generation channels in the photoinduced electron transfer process. The ratio of ultrafast carrier generation is 53%, while the portion of ultrafast carrier generation is 10% with addition of CN which acts as a solvent additive. Figure 12f shows the TA spectra of poly[(4,4‐bis(2‐ethylhexy)dithieno[3,2‐*b*:2′,3′‐*d*]silole)‐2,6‐diyl‐*alt*‐(4,7‐bis(2‐thienyl)‐2,1,3‐benzothiadiazole)‐4,7‐diyl] (PSBTBT) pristine (broken line) and PSBTBT/[6,6]‐phenyl‐C_61_‐butyric acid methyl ester (PCBM) mixed films prepared from *o*‐dichlorobenzene.[[qv: 88c]] Ohkita et al. took the TA spectroscopy measurement in the excitation of PSBTBT domains electively with excited wavelength at 800 nm. This measurement investigated the dynamics of singlet excitons generation from PSBTBT. The TA spectra of PSBTBT pristine show a broadband absorption at around 1500 nm and decayed monotonically with time, which was attributed to the absorption of PSBTBT singlet excitons upon excitation.^100^ The singlet exciton signals around 1500 nm decayed in several ps. The broadband absorption region from 800 to 1300 nm gradually decays with increasing time. The singlet exciton band disappeared entirely at 10 ps after laser excitation and the broadband around 1300 nm decayed in a hugely different time more than 1 µs. These discoveries suggest that these two absorption regions are different species. Obvious evidence is shown in Figure 12g, the broadband absorption region around 1300 nm decayed in several microseconds. This is obviously distinct from PSBTBT triplet excitons, which have an absorption peak around 1100 nm.^101^ Thus, these absorption bands around 1300 nm are attributed to PSBTBT polarons.

The TA measurement of excitation electron transfer in the DSSC system is an effective method for understanding the dynamics of electron/energy transfer processes. These results can be expected to have direct ramifications for enhancing the performance of DSSC and opening a new generation for solar energy conversion devices.

## 5 Conclusions and Perspectives

TA spectroscopy is a powerful technique for detailed time‐resolved studies of the electron transfer dynamics occurring in the nanostructured materials/organic molecular hybrid systems. The fundamental photochemical and photophysical processes of the interfacial area within nanostructured materials and optoelectronics applications, including photovoltaic and photocatalysis, could be demonstrated by TA spectroscopic technique. The temporal scale of the interfacial charge transfer dynamic processes of optoelectronic applications ranges from the ps to the ms. This review presented a brief summary of direct observation of the interfacial charge transfer among semiconductor nanomaterials and organic complexes. The photoinduced excitons can be transferred to the corresponding energy levels of organic complexes through excited‐state energy transfer procedures including SSET, TTET, and TTA. The transient kinetics of excited states generation, separation, and transfer are able to be measured by TA spectroscopic techniques. Understanding the transient kinetics is beneficial to the design rationality of optoelectronic applications. We have also provided a compendious discussion of the charge transfer dynamics in photocatalytic systems and solar cells. In these optoelectronic applications, the dynamic parameters of excited states from the donors and acceptors can be observed intuitively via TA spectroscopy to show the constant of the reaction rate in photocatalysis and the photoelectric conversion rate in the solar cell, so as to improve the performance of these optoelectronic applications.

To date, TA spectroscopy has been utilized in an impressive range of photophysical and photochemical systems to reveal the distinct role of photoinduced excited states for controlling the kinetics of energy conversion. Future applications of this technique will be benefited from the ongoing developments in detector technologies, femtosecond and nanosecond laser sources, nonlinear optical frequency conversion schemes, as well as synchrotron and free electron laser accelerator physics.

Although ultrafast TA spectroscopy has been demonstrated as a promising approach to investigate the ultrafast dynamics of charge transfer and photochemical reactions in solar energy conversion, there are still lots of other excellent technologies which focus on the development of optical materials as well as their heterostructured hybrid systems. Therefore, the major limitations and challenges of the ultrafast time‐resolved spectroscopy techniques are how to combine the fundamental photoinduced charge transfer processes with the overall devices or optoelectronic applications. Specifically, further efforts should be made for studying the in situ processes of the solar energy conversion in these optoelectronic devices, which can provide guidelines for the development of low‐cost and high‐efficiency photovoltaic technologies. On the other hand, the charge carrier dynamics at the interfaces of nanostructured materials are more complicated than the molecules systems. In most cases, the ultrafast TA measurements of nanostructured materials or device systems are approximated into the molecule reactions. While the surface physics of nanomaterials and optoelectronic devices are significantly different from the molecular photophysics. Therefore, it is a critical challenge to develop an ultrafast time‐resolved spectroscopy method to study the surface electronic structures and corresponding dynamics in solar energy conversion devices.

## Conflict of Interest

The authors declare no conflict of interest.
